# Registered Nurse to Bachelor of Science in Nursing: nesting a fast-track to traditional generic program, teachings from nursing education in Burkina Faso

**DOI:** 10.1186/s12912-015-0118-2

**Published:** 2015-12-02

**Authors:** Idrissa Beogo, Chieh-Yu Liu, Colile P. Dlamini, Marie-Pierre Gagnon

**Affiliations:** 1 École Nationale de Santé Publique, Ouagadougou, Burkina Faso; 2 Faculté des sciences infirmières, Université Laval, Pavillon Ferdinand-Vandry, 1050, Avenue de la Médecine, Québec, Québec G1V 0A6 Canada; 3 School of Nursing, National Taipei University of Nursing and Health Sciences, Taipei, Taiwan; 4 University of Swaziland, Faculty of Health Sciences, Mbabane, Swaziland

**Keywords:** Burkina Faso, Nursing education, Nested fast-track, Accelerated program, Pre-registered, Post-registered

## Abstract

**Background:**

Nursing education has evolved over time to fit societies’ increasing care needs. Innovations in nursing education draw thorny debates on potential jeopardy in the quality, safety, and efficacy of nurse graduates. Accelerated nursing education programs have been among landmark strategic changes to address the persistent bedside nurse shortage. Despite the dearth of empirical studies in sub-Saharan Africa (SSA), the National School of Public Health of Burkina Faso has developed a State Diploma Nursing (SDN) fast-track program. With innovative features, the program is nested into the traditional SDN program. This study investigates preliminary outcomes of the implemented policy using the initial cohort that went through the program. Comparison of the traditional generic program and the fast-track one is drawn to inform nursing education policy.

**Methods:**

The study was conducted in the three campuses delivering the SDN program. Data collected from a representative sample included 255 students from the 2006–2009 cohort, after concluding the program. Surveyed students were assessed according to the program entry status. Outcomes were measured using students’ academic performance. Besides descriptive analysis, bivariate *t*-test, F-test, and multivariate ordinary least square regression (OLSR) were employed to determine the comparative pattern between the traditional generic and the newly nested fast-track program. Students’ varied statuses (private pre-registration, state pre-registration, private post-registration, and state post-registration) were kept to better outline the findings trend.

**Results:**

A fifth (19.6 %) of surveyed students were enrolled in the fast-track stream from which, one third (33.7 %) consisted of post-registered students. Fast-track students comparatively achieved the best academic performance (mean: 73.68/100, SD: 5.52). Multivariate OLSR confirmed that fast-track students performed better (β: 5.559, *p* < 0.001), and further informed differences between campuses. Students entry status also displayed significant differences, yet the academic performance of post-registered students from traditional generic versus fast-track was similar (*p* = 0.409).

**Conclusion:**

Findings suggest that fast-track program students performed better than the ones from the traditional generic program. The uniqueness and success of this mixed nursing program experience sheds light for nursing educators engaged in policy making. The study results can serve as a crucial foundation for policymakers to alleviate the nurse shortage in SSA.

## Background

Nursing education has demonstrated a phenomenal growth in term of program numbers, variety or content delivered [1]. Despite the contentious characteristics of any innovation, nursing education has evolved over time in response to societal trends, and increasing healthcare demands. The challenges fueling thorny and endless debates on innovative approaches prompted in nursing education serve to insure that professional competence standards are maintained. One of the landmark innovations is based on accelerated nursing education programs developed to tackle the persistent shortage of nurses. The accelerated (or fast-track in other literature) program is described as any nursing education program where the standard length of traditional generic curriculum is significantly shortened [2].

This concern affects the Masters of Science in Nursing (MSN) programs and especially the Bachelor of Science in Nursing (BSN) [3]. The literature reveals an array of options: licensed practical nurse to registered nurse (RN), accelerated BSN degree program [2, 4] non-nursing college graduate to MSN [5, 6] second degree accelerated BSN program [7–9], distance accelerated program [10] or fast-track back or refresh program [11–13]. A wealth of nursing education literature informs us on various pedagogic strategies that are implemented in high income countries, while in sub-Saharan Africa (SSA), despite the nursing education scope and endeavors to work at the forefront, very little has been published. The present study represents an attempt to assess the development and refinement of the fast-track program recently implemented in Burkina Faso, drawing upon established research-based evidence on this subject in SSA.

### Fast-track programs and nursing competence outcomes

There is an established shortage of adequately trained professionals worldwide, including in resource-constrained settings like Burkina Faso [14]. This shortage threatens healthcare systems as nurses form the majority of the healthcare workforce [15, 16] and deliver most healthcare services [16]. Accelerated programs experience exponential growth [17–20] with the intent to meet short-term needs [21], yet attract more students [22] including the untapped reservoir of learners [4]. This is true for resource constrained settings like Burkina Faso where experienced assistant degree nurses (ADN) (herein French denomination, Infirmier/Infirmière Bréveté) before 2006, sat for a 3-years State Diploma Nursing (SDN) program (herein French denomination, Infirmier/Infirmière Diplomé d’État). Shortening the training length has been viewed as a solution to the workforce shortage problem [14] and led to an exponential growth of fast-track programs over the last 2 decades [17, 23], saving time and money. These programs are intense, fast paced and recruit students typically different from those sitting for the traditional baccalaureate programs [7]. The average length of a BSN program is 12 to 18 months [21, 24]. It attracts adult learners because its structure and mode of instruction is developed to meet their learning needs and ensures equity by recognizing their prior learning and field experiences [1].

The concept of fast-track program dates back to the 1950s, when an ADN education program was developed to meet short-term system needs of the post World War II United States (US) [25]. It was conceived to reduce the training period of nurses to tackle the then abrupt shortage caused by multiple factors which included increased needs for healthcare services related to discoveries in terms of antibiotics and progress in terms of surgery [26], infrastructure extension [27], and the siphoning of nurses for the war effort on the battlefield [28]. Such a program was in the same category as the Physician Assistant (PA) and Nurse Practitioner (NP) designed in 1965 for a doctor fast-track curriculum to respond to a shortage of physicians [29]. Internationally, fast-track in nursing education was later implemented for costs containment purposes [30]. Its success led to replication in New Zealand, Australia, Ireland [29, 31, 32], the UK and Canada [8, 33] and Taiwan [34]. Burkina Faso has experienced running a SDN fast-track program (not nested), especially designed for post-registered associate degree nurses. For unknown reasons –probably political– only two cohorts were trained from 1988 to 1991.

Studies comparing the traditional generic students’ performance to accelerated ones showed similar to better performance of the latter group. Some studies, however, did not find differences between fast-trackers and traditional generic students’ grades [35]. Nevertheless, Bentley [36] asserted that the performance of students in the accelerated program was just as good as those in traditional program, while Lee-Hsieh [34] in Taiwan or Orsolini-Hain and Waters in the US [26] have all established that fast-track students did similarly or outperformed traditional program’s students. In one follow-up study, participants’ perception significantly increased for all items assessed a year of service after graduation [37].

### Nursing programs and entry venues in Burkina Faso

Father Goarnisson pioneered nursing training in Upper Volta (current Burkina Faso). The first formal nursing school, the National School of Nursing, was founded in 1968 [38] and upgraded into the actual format, National School of Public Health (NSPH) (herein French denomination, École Nationale de Santé Publique) in 1977 [39]. Two nursing programs are provided. First, a 2-year ADN program, referred elsewhere as enrolled nursing program [40, 41]. The second is the SDN, a 3-year program widely known as the traditional generic program equivalent to BSN in common literature. The ADN candidates hold 10 years of general education, while SDN program requires at least 13 years. SDN curriculum is oriented towards the medical model and divided into six areas (basic sciences, nursing profession, men and women’s health, infant and youth health, community health, health service management), focusing on the nursing care and management of diseases and conditions. This curriculum is designed so that graduates are competent to practice with minimal supervision, to hit the road running.

As elsewhere [42], ADN programs are supposed to prepare “technical” nurses, whereas BSN programs –preferred entry level for professional nursing practice [20]– prepare “professional” nurses skilled in leadership/management, community/public health, nursing research, evidence-based practice, and professionalism/legal and ethical issues. Of the 6196 nurses reported in Burkina Faso in 2013, some 43.7 % were ADN graduates [43]. The NSPH, by far the main training school, graduates approximately 350 state diploma nurses on annual basis. Before the market liberalization (1991), the government was the only employer and the NSPH was the only trainer. Since then, the private sector has become involved in training and in healthcare and services provision [44]. Therefore, in addition to the aforementioned status, the NSPH enlists private pre-registered and post-registered students according to a quota. The following are the avenues to SDN program:

1. Non-nurse candidates looking for government position may sit for a national test. Successful candidates attend the SDN program as state pre-registered students, granted a stipend, waived from paying tuition, and serve the government upon completion. Candidates applying privately are given the status of private pre-registered students.
2. State ADN may enter the SDN program after being shortlisted in the national test. Five years of experience as ADN is a pre-requisite. They are labelled state post-registered students. Under the new scheme, students attend the fast-track program for two calendar years and because of the nested feature, they directly join the SDN program in their sophomore year.
3. Non-government ADN may apply for the SDN program as private post-registered students. So far, there is a vacuum in the selection policy; their recruitment to the program is based on merit.

Students sit for a summative examination at the end of both year 1 and year 2. The third year summative examination serves as a graduation examination. This arrangement is similar to what is done in Norway and in Australia [45] and in some SSA countries like Botswana, Swaziland, Zimbabwe [40] unlike in the US where the NCLEX is run. Controversies about the admission policy into nursing programs continue to rock nursing education [46]. The question is whether to accept only high-achieving candidates or to take a risk by recruiting those with a deep desire to become nurses but do not meet admission requirements. Newton et al. [47] claimed that admission policies to foster BSN candidateship can either promote success or facilitate failure. Except “good pre-nursing” preparedness, there is no hard evidence on the best policies between traditional “ranked”, multiple points or rolling admission [46]. Students with a weak academic level can benefit from support programs especially designed to retain students and help them graduate [48].

The innovative fast-track in Burkina Faso nursing education is unique. The current program contains features that are not common in the literature. In the nested scheme, students attend the same classes, practicum, and rural setting internship in the third year. The fast-trackers are not required to take any transitional courses but must pass a paper-pencil test. ADN to SDN fast-track program prevents useless repetition [34] as students learn basic nursing skills during the initial year. The Burkina Faso model is distinct in that it combines two tracks in the same classroom from the sophomore year.

While accelerated programs have proven to be at least as effective as the traditional generic programs in industrialized countries, very little is known about the implementation of such initiative in SSA such as the fast-track SDN program implemented in Burkina Faso. This study intended to achieve the following objectives: 1) to evaluate the performance of fast-track students of the 2006–2009 cohort, 2) to compare the performance of students in the traditional generic program to those in the fast-track SDN program, and 3) to draw a conclusion on whether the fast-track policy is justified.

## Methods

The study was carried out at NSPH, which is a government-owned institution with six outlying campuses. The three campuses that offer the SDN program were included: Ouagadougou, the main campus and two outlying campuses, Bobo Dioulasso and Ouahigouya. We conducted a quasi-experimental survey design with a descriptive and comparative approach. Participation comprised of 255 students who were blindly captured from a total of 356 who formed the 2006–09 cohort. This cohort was the first to include a fast-track program. Only three students failed and were excluded from participation. Of these 255 students, 50 attended the fast-track program (2-year SDN program) while 205 were registered in the traditional generic program (3-year SDN program). Among the latter, 36 were post-registered (last batch of post-registered enrolled in 3-year SDN program).

All students, from both the fast-track and generic programs, were invited to take part in this study through an announcement posted at the school. Students who chose to participate were informed in detail on the nature of the study, their rights and responsibilities, and were asked to sign a participation agreement form. Data were collected in two phases. On phase one, students were asked to fill out a self-administrated questionnaire. Demographic, socio-economic and achievements data were gathered from both generic and fast-trackers. For phase two, the Principal Investigator (PI) (BI), conducted a document review from the campuses’ academic affairs, to solicit all students’ academic scores (course-work and year-end exam scores). In order to protect participants, no identifier was assigned. Collected data were restricted to information required for performance analysis for the purpose of the study.

### Survey instrument and variables

The instrument employed was derived from the Clinical Nursing Competence Questionnaire [34]. It is a structured self-administered questionnaire with three parts: The first section of the questionnaire consisted of socio-demographic variables (birth date, gender, marital status, number of children). The second section included academic information: campus, registration status, and track. Finally, the last section sought detailed information on grades by module and academic year: 1) paper-pencil scores, 2) clinical practicum scores, 3) summative school scores (paper-pencil and clinical practicum, on 50 % basis) plus year-end examination scores (for both year 1 and 2, weighing equally), and, 4) third year (graduation) scores. The latter consisted of the summative school and graduation examination scores. Corresponding weight was applied to each module. For instance, in third year, each of the summative school and graduation examination score counted for 50 %. In breaking down the latter, nursing clinics examination accounted for 30 % of the score. It is approximately 20 % for year-end examinations and varies for practicum, depending on the number of sessions achieved over the academic year. For each academic year, we created sub-items summing up the theoretical examinations and practicum sessions (simulation and bedside practicum). To that, the internship in a rural setting was added into the third year. All scores were computed and reported to a 100 point-scale, the success rate being 60 %.

The questionaire has undergone a content validation, forward from English to French and backward as translated by a professional translator. Five experienced English teachers from the English department of the University of Ouagadougou in Burkina Faso, worked independently, examining every step of the translation process.

The pilot test was conducted between June 16th and 30th, 2009 and included 10 nursing students from Bobo Dioulasso, and 10 others from the Ouagadougou campus, using a convenient sampling. Participants were asked to complete the form impromptu. The response rate was 100 %. Minor adjustments occurred for demographic items such as the structure of the birth data, some items were reworded. The pilot test was run to ensure better ways of presenting the tool items for more accurate responses and ratings.

The dependent variable consisted of students’ academic performance stratified by track. The explanatory variables were categorized into demographic and academic variables. As indicated in Table 1, the variables age, registration status and campus were dummied.

**Table 1:** Variables definition

Dependent variables	
Score achieved	Year1, Year2 and Year3 performance Year2 & 3 sum up
Explanatory variables	
Demographics	
Age, year	Two dummy variables: 0: 20–29, 1: 30–39, 2: 40 & more
Gender	0: for male, 1: otherwise
Marital status	0: Married (formal + engaged), 1: Single
Number of children	0: childless, 1: otherwise
Academic variables	
Registered track	0 Traditional generic program, 1 Fast-track program
Registration status	Four dummy variables: 0 State pre-registration, 1 Private pre-registration, 2 State fast-track registration, 3 Private fast-track registration, 4 3-year state post-registration
Campus	Two dummy variables: 0 Bobo Dioulasso campus, 1 Ouagadougou campus, 2 Ouahigouya campus

### Ethical considerations

The study was first authorized by the National Taipei University of Nursing and Health Sciences Institutional Review Board (IRB#: 98A214 of June 25, 2009). Furthermore, we obtained authorization from the NSPH headquarters and the each campus principal, so that the academic affairs department from campuses could allow data access to the PI for on-site use. The participation was completely voluntary, anonymous, and freedom to withdraw in the process was guaranteed. Before the survey questionnaire was administrated, every participant provided signed written informed consent.

### Data analysis

This survey employed a three-stage analytic design. First, participants were described by track. The measures of central tendency were computed for each group. Next, group comparisons were examined using 2-sample *t*-test and ANOVA. Finally, a multivariate analysis was conducted using ordinary least square regression (OLSR). We assumed equivalence among students of post-registered status. Therefore, to figure out the true effect of track, we excluded the post-registered students from the generic program from the multivariate analysis. They could have been fast-trackers if the policy was implemented a year earlier, or if they had entered a year later. Data were entered without personal identifiers and analyzed with SPSS 18 for Windows. The two-tailed was tested with 95 % confidence level (*p*-value at <0. 05) considered statistically significant.

## Results and discussion

This study on the fast-track program nested in the traditional generic SDN program is a baseline assessment of a project aiming to provide evidence concerning the policy’s worth. This assessment targeted the first cohort (2006–2009) that was unique in many ways. First, it includes both the last batch of post-registered students who sat for the 3-traditional generic SDN program and the first intake of fast-trackers. The two groups are used as proxies of the former system and the new policy implemented, respectively. Assessment of this group allowed a critical analysis between comparable subsamples. Second, all three campuses offering the program at NSPH were committed to its implementation. Finally, this initial assessment served to evaluate the policy in order to inform future developments of the program. Of the 255 students participating, one out of 5 (*n* = 50) attended the fast-track, 42 were post-registered students attending the 3-year SDN program (the last group). Two-third were male, while half of the students were under the age of 30 (*n* = 129). The majority (58 %) of students were from the main campus (Ouagadougou) (Table 2). Table 3 provides a comparison pattern between the two tracks. For instance, most of the traditional generic students were less than 30 years old, 61.5 versus 6.0 % among fast-trackers; *t*-test shows an average of 7.5 more years for the latter group (*p* < 0.001) (not shown). Elsewhere, accelerated program students tend to be the oldest [1, 49]. Unsurprisingly, in Burkina Faso, the fast-track program is designed for nurses who have been working awhile.

**Table 2:** Characteristics of studied nursing students

Variables	Frequency	
	*n*	%
Gender		
Male	174	68.2
Female	81	31.8
Age, year		
20–29	129	50.6
30–39	103	40.4
40 & above	23	9.0
Campus location		
Bobo Dioulasso	68	26.7
Ouagadougou	148	58.0
Ouahigouya	39	15.3
Track		
Traditional generic	205	80.39
Fast-track	50	19.6
Registration status		
Traditional Generic		
State pre-registration	103	40.4
Private pre-registration	66	25.9
3-year state post-registration^a^	36	14.1
Fast-track		
State Fast-track registration	42	16.5
Private Fast-track registration	8	3.1
Marital status		
Married & engaged	111	43.5
Single	139	54.5
Missing	5	2.0
Number of children		
Childless	140	54.9
1 child	104	40.8
Missing	11	4.3

^a^Last batch of post-registered students completing the traditional generic track (3-year program)

**Table 3:** Profile of generic program compared to fast-track students

Variables	Traditional generic track		Fast-track	
	*n*	%	*n*	%
Gender				
Male	134	65.4	40	80.0
Female	71	34.6	10	20.0
Age, year				
20–29	126	61.5	3	6.0
30–39	66	32.2	37	74.0
40 & above	13	6.3	10	20.0
Campus location				
Bobo Dioulasso	64	31.2	4	8.0
Ouagadougou	110	53.7	38	76.0
Ouahigouya	31	15.1	8	16.0
Registration				
State pre-registration	103	50.2	–	
Private registration	66	32.2	8	16.0
Post-registration	36	17.6	42	84.0
Marital status				
Married & Engaged	75	36.6	36	72.0
Single	125	60.9	14	28.0
Missing	5	2.5	–	
Number of children				
Childless	127	62.0	13	26.0
≥ 1 child	67	32.7	37	74.0
Missing	11	5.3	–	

For comparability between tracks, analyses included students’ performance achieved from sophomore year. Table 4 unveils significant statistical differences within tracks. In traditional generic tracks, post-registered students’ performance surpasses that of pre-registered students (F: 26.402, *p* < 0.001). Between tracks differences show that fast-trackers have 5 points more than traditional generic students (*t*: −5.86, *p* < 0.001).

**Table 4:** Compared performance^a^ between tracks by selected variables

Variables	Traditional generic track (*n* = 205)				Fast-track (*n* = 50)			
	Performance/100 points baseline				Performance/100 points baseline			
	*n*	Mean	SD	F/t (*p*-value)	*n*	Mean	SD	F/t (*p*-value)
Gender								
Male	134	70.48	6.01	−2.989 (0.003)	40	74.54	5.24	−2.287 (0.027)
Female	71	68.04	5.30		10	70.25	5.51	
Age, year								
20–29	126	68.99	5.41	2.055 (0.131)	3	68.88	3.56	2.924 (0.064)
30–39	66	70.54	6.71		37	74.71	5.43	
≥ 40	13	71.26	5.16		10	71.29	5.14	
Registration								
State pre-registration	103	69.72	5.24	26.402 (<0.001)	–			
Private pre-registration	66	66.76	5.13		–			
3-year state post-registration	36	74.68	5.52		–			
Private FT post-registration	–				8	69.75	6.43	5.226 (0 0.027)
State FT- registration	–				42	74.43	5.08	
Tracks^b^	169	68.56	5.38		50	73.68	5.52	−5.86 (<0.001)
Campus								
Bobo Dioulasso^0^	64	67.62	4.88	7.296 (0.001)	4	75.12	1.68	0.279 (0.758)
Ouagadougou^1^	110	70.11	6.15	2 = 1 < 0^c^	38	73.36	5.62	
Ouahigouya^2^	31	72.11	5.61		8	74.49	6.52	
Gender								
Male	134	70.48	6.01	−2.989 (0.003)	40	74.54	5.24	−2.287 (0.027)
Female	71	68.04	5.30		10	70.25	5.51	
Number of children								
Childless	127	69.02	5.53	−2.028 (0.044)	13	75.46	6.73	1.364 (0.179)
≥ 1 child	67	70.82	6.49		37	73.05	4.98	

^a^2 years performance (sophomore & 3^rd^)

^b^Post registered students (*n* = 36) of traditional generic program is dropped

^c^Bonferroni Post Hoc Test: ^0^Bobo Dioulasso campus, ^1^Ouagadougou campus, ^2^Ouahigouya campus

FT: fast-track

In the regression model, gender, marital status, and number of children did not reveal any statistical association. Because of family strain impact, this absence of difference to a certain extent violates the common perception that accelerated programs are notoriously intense, therefore demanding. Earlier studies pinpointed challenges like family, friends, and leisure foregoing [9] as affecting the learning process of adult learners.

The assessment of track and registration status into the model led to a higher correlation (*r*: 0.844, *p* < 0.001), underpinning a high collineality. We therefore ran two models. The first integrated registration status to distinguish between pre-registered (state and private). The fast-trackers, on average, have 4.5 points more than generic state registered students (β = 3.630, *p* < 0.001). In model 2, fast-trackers performed significantly better than generic track students (β = 5.559, *p* < 0.001). This finding confirms evidence from earlier studies on pure performance [24, 50] and professional values [51]. Thus, the American Association of Colleges of Nursing reported that accelerated program students are high-achieving, motivated learners, do well on the NCLEX, and are valued by many employers even as new graduates [5, 52, 53]. Others contend that since their previous experience matters [3], they come to this educational program with a wealth of experiences and knowledge [7, 24]. Finally, as a concluding statement Kowitlawakul et al. [21] asserted that registration status is a reliable predictor of academic performance within nursing program.

We further tested the post-registered students from the generic program versus the fast-track students; which served as a sensibility analysis. We found no significant difference (*t* = 0.830, *p* = 0.409, 95 % CI = −1.399–3.403). The comparison of their annual score suggested a similar trend (Table 5). This absence of difference suggests that post-registered students attending the SDN program either for 3 years (traditional generic program) or for 2 years (fast-track) perform alike, which indicates the worth of the fast-track policy (Table 6).

**Table 5:** Compared performance of post-registered students with the generic & fast track stream

Academic performance	Track	*n*	Mean	SD	95 % CI
Year2 performance	Traditional generic	36	74.86	6.946	0.852 (−2.433–2.938)
	Fast-track	50	74.61	5.567	
Year3 performance	Traditional generic	36	74.49	4.633	0.135 (−0.556–4.059)
	Fast-track	50	72.74	6.126	
Sum of Y2 & Y3 score	Traditional generic	36	74.68	5.528	0.409 (−1.399–3.403)
	Fast-track	50	73.68	5.521

**Table 6:** OLSR of predictors of State Diploma Nurses’ performance of nested fast-tract program versus traditional generic program

Variables	Model 1			Model 2		
	β (SE)	*t*	CI	β (SE)	*t*	CI
Age, year						
20–29 (Ref.)	**1**			**1**		
30–39	−0.377 (0.966)	−0.391	−2.281–1.527	−0.682 (0.967)	−0.706	−2.590–1.225
≥ 40	−1.652 (1.942)	−0.851	−5.481–2.177	−1.754 (1.963)	−0.894	−5.624–2.116
Gender						
Male (Ref.)	**1**			**1**		
Female	1.014 (0.903)	1.123	−0.767–2.796	1.874* (0.833)	2.250	0.232–3.517
Marital status						
Single (Ref.)	**1**			**1**		
Married	−0.101 (1.024)	−0.099	−2.121–1.918	0.010 (1.034)	0.009	−2.030–2.049
Number of children						
≥ 1 child (Ref.)	**1**			**1**		
Childless	−0.895 (1.101)	−0.813	−3.065–1.275	−0.892 (1.113)	−0.802	−3.087–1.302
Campus location						
Bobo Dioulasso (Ref.)	**1**			**1**		
Ouagadougou	2.001 (0.898)	2.227*	0.229–3.772	1.653 (0.896)	1.845	−0.114–3.419
Ouahigouya	3.733 (1.127)	3.312**	1.510–5.956	3.695** (1.139)	3.243	1.448–5.942
Registration status						
State Pre-Registered (Ref.)	**1**					
Private Pre-Registered	−2.276 (0.980)	−2.321*	−4.209–(−)0.342	–	–	–
Fast-track	4.506 (1.242)	3.630***	2.058–6.955	–	–	–
Track						
Traditional generic (3-year) (Ref.)	–	–	–	**1**		
Fast-track (2-year)	–	–	–	5.559*** (1.169)	4.756	3.254–7.863

*p < 0.05

**p = 0.01

***p < 0.001

Ref.: reference group

β: coefficient

SE: standard error

t: t-statistic

CI: confidence interval (95 %)

Model 1: considers the registration status. State Pre-Registered is used as the reference group against Private Pre-Registered and Fast-track

Model 2: includers the track variable. Traditional generic (3-year) is used as the reference group against Fast-track (2-year)

Clearly, students from the fast-track program performed better than their generic program counterparts. A side-by-side comparison demonstrates that the group of post-registered students who attended the generic program (State post-registered) also performed significantly better than those who were pre-registered (State and private) (Table 4). This hierarchy in performance draws two principal teachings: first, Lee-Hsieh et al. [34] thought students re-entering a nursing academic environment were seen to display maturity, more devotion, and motivation in studies. The better performance of the post-registered students and the insignificant difference between fast-trackers and the other post-registered students from traditional generic track (Tables 4 and 5) could rationally justify the fast-track program. Secondly, the weak scores of private pre-registered students might not be surprising. While state pre-registered students enter through a stringent test, approximately only 12 % passed the enrollment test which is officially required in the NSPH [54]. In the same study, private registered students were seen to be lacking motivation; viewing the program as a stop gap or pushed by parents to apply. Nevertheless, it is difficult to prevent the program from admitting ill-prepared or uninterested students. For Newton et al. [47], though the rolling admission policy aims to enhance students’ recruitment by filling admission quotas, quality is somehow compromised. Moreover, a number of studies associate weak pre-nursing grades to insufficient outcome [55].

## Conclusion and recommendations

This mixed program is unique in literature and the present work is a baseline assessment of the innovative nested fast-track SDN program implemented in Burkina Faso. It compared the performance of students enrolled in nested fast-track programs introduced by the NSPH, being the first of its kind in nursing education literature. As the baseline assessment, this single cohort-focus study has the unique merit of gathering the initial fast-track batch, and the last group of post-registered attending the 3-year traditional generic program. Our findings suggest the performance standing for the upcoming cohorts, provide the basis for future studies, and finally inform appropriate policy options. We can acknowledge the plausibility of findings as participants took the same courses within the same educational context and conditions for comparability. A significant difference between tracks and the similarities among post-registered students confirmed that fast-trackers’ academic performance was considerably better, justifying the worth of its implementation. This unique, but progressive example may inspire other nursing schools, especially from SSA to reform their programs to suit their contextual needs and resources.

Findings show significant differences between the campuses in the same school, raising critical questions in the program delivery, student prerequisites, and co-requisites. From these important differences within the same institution, we can assume that the discrepancy may exist and may even be huge with other nursing schools in the country (all private). The NSPH has more than 40 years of history and is thus experienced in nursing education, administration, and clinical placement networks. As students’ educational quality declines [56] in the absence of quality assurance mechanisms, we suggest –although still questioned [45]– that an entry examination is essential to ensure that nurses are competent upon registration [57]. Furthermore, applying a State licensing system would push schools to become pro-active and promote higher achievement and shore up employers’ confidence since they want graduates who are ready [58].

This study had some strengths and limitations. First, study participants shared the same learning environment, which made it possible to control most external factors that could have influenced their performance. Second, the sample size was large enough and included all registration statuses codified in the country. Though our study used grades, including clinics, some criticized a certain reductionism when approaching clinical nursing competence [45]. Finally, participants in this study consisted of one cohort (2006–2009) and were from one institution (three campuses), making it difficult to generalize findings. Large scale studies, involving varied designs, including several nursing schools and other stakeholders (clinical institutions, employers, etc.) are necessary to ascertain present conclusions and strengthens the evidence supporting the success of the policy. Nevertheless, our findings provide foundational evidence that the fast-track nursing program is meaningful, particularly for resource-constrained settings.

### Terminology used

*Registration status*: academic designation defining the level or position or prior background of candidate-students while she/he is being enrolled in the nursing program.

*Pre*-*registered*: are novice students entering into the SDN program without any prior background in nursing.

*Post*-*registered*: are registered ADN re-entering into the nursing program to equip themselves with further specialized practice and knowledge. In Burkina Faso, those candidates must hold 3 to 5 years of bed-side experience.

*State registered students*: are students recruited into the program through the mechanism of a national test. They are provided a stipend, exempted from paying any charge related to registration, tuition and are systematically employed by the government on completion.

*Private registered students*: they are either novice (pre-registered) or re-entering nursing education (post-registered) and required to pay tuition fees.

*Academic performance*: is the measurable outcome of scholastic achievement, shown through test scores and total course points earned.

*Fast*-*track program*: Accelerated nursing program taken for two calendar years upon an ADN program completion.

*Traditional generic program*: is the traditional 3-year nursing program attended by new non-nurse applicants.
